# Characterization of Synonymous *BRCA1*:c.132C>T as a Pathogenic Variant

**DOI:** 10.3389/fonc.2021.812656

**Published:** 2022-01-11

**Authors:** Jun Li, Ping Wang, Cuiyun Zhang, Sile Han, Han Xiao, Zhiyuan Liu, Xiaoyan Wang, Weiling Liu, Bing Wei, Jie Ma, Hongle Li, Yongjun Guo

**Affiliations:** ^1^ Department of Molecular Pathology, The Affiliated Cancer Hospital of Zhengzhou University and Henan Cancer Hospital, Zhengzhou, China; ^2^ Henan Key Laboratory of Molecular Pathology, Zhengzhou, China; ^3^ Henan International Joint Laboratory of Cancer Genetics, Zhengzhou, China; ^4^ Department of Pathophysiology, School of Basic Medical Science, Zhengzhou University, Zhengzhou, China; ^5^ Department of Breast Surgery, The First Affiliated Hospital of Zhengzhou University, Zhengzhou, China; ^6^ Amoy Diagnostics Co., Ltd. (AmoyDx), Xiamen, China; ^7^ Department of Medical Oncology, The Affiliated Cancer Hospital of Zhengzhou University and Henan Cancer Hospital, Zhengzou, China

**Keywords:** *BRCA1/2*, splicing variants, HBOC, variants classification, synonymous variants

## Abstract

Breast cancer gene 1 *(BRCA1)* and *BRCA2* are tumor suppressors involved in DNA damage response and repair. Carriers of germline pathogenic or likely pathogenic variants in *BRCA1* or *BRCA2* have significantly increased lifetime risks of breast cancer, ovarian cancer, and other cancer types; this phenomenon is known as hereditary breast and ovarian cancer (HBOC) syndrome. Accurate interpretation of *BRCA1* and *BRCA2* variants is important not only for disease management in patients, but also for determining preventative measures for their families. *BRCA1*:c.132C>T (p.Cys44=) is a synonymous variant recorded in the ClinVar database with “conflicting interpretations of its pathogenicity”. Here, we report our clinical tests in which we identified this variant in two unrelated patients, both of whom developed breast cancer at an early age with ovarian presentation a few years later and had a family history of relevant cancers. Minigene assay showed that this change caused a four-nucleotide loss at the end of exon 3, resulting in a truncated p.Cys44Tyrfs*5 protein. Reverse transcription-polymerase chain reaction identified two fragments (123 and 119 bp) using RNA isolated from patient blood samples, in consistency with the results of the minigene assay. Collectively, we classified *BRCA1*:c.132C>T (p.Cys44=) as a pathogenic variant, as evidenced by functional studies, RNA analysis, and the patients’ family histories. By analyzing variants recorded in the BRCA Exchange database, we found synonymous changes at the ends of exons could potentially influence splicing; meanwhile, current *in silico* tools could not predict splicing changes efficiently if the variants were in the middle of an exon, or in the deep intron region. Future studies should attempt to identify variants that influence gene expression and post-transcription modifications to improve our understanding of *BRCA1* and *BRCA2*, as well as their related cancers.

## Introduction

Hereditary breast and ovarian cancer (HBOC) syndrome is an autosomal dominant genetic disorder that is caused by the predisposition of pathogenic variants in breast cancer gene 1 (*BRCA1*) and *BRCA2*. It is characterized by an increased lifetime risk of developing breast cancer, ovarian cancer (OC) and prostate cancer, as well as other cancers to a lower extent, such as pancreatic cancer (1). According to the National Comprehensive Cancer Network guidelines for 2021, identifying heterozygous pathogenic *BRCA1/2* germline variants (g*BRCA^MUT^*) in affected individuals is sufficient for the diagnosis of HBOC. This test is of paramount importance for both patients and their families, as knowing the g*BRCA^MUT^* status can not only guide the treatment options for patients, but also help carriers to take preventive measures. The commonly used variant classification criteria were drafted by experts from different academic committees (2–4); in general, a five-tier classification system [Benign, Likely Benign, Variant of Uncertain Significance (VUS), Likely Pathogenic, and Pathogenic] is used to interpret detected variants to guide therapeutic decisions and disease management. However, it is particularly difficult for oncologists and genetic counselors to deal with variants classified as VUS (5).

Efforts to reduce the number of VUSs in *BRCA1/2* genetic testing have been ongoing for decades, including classical function studies to evaluate the biological consequences of specific variants (6), clustered regularly interspaced short palindromic repeats (CRISPR)/Cas9-based saturation genome editing of all possible single nucleotide variants (SNVs) in a defined region to test their effects in the native genomic context (7), yeast 2-hybrid minigene assays to determine splicing changes (8), *in silico* predictions of changes in protein structure and function (9), and multifactorial likelihood quantitative analysis (10), among others. These methods have been systematically reviewed by several researchers (11–13).

However, approximately 7–10% of variants detected in *BRCA1/2* are still classified as VUSs (14–16), depending on the criteria used for classification and the training of staff in different laboratories. Most VUSs in *BRCA1/2* are missense variants, which have uncertain influences on the function of the protein product and the cancer risk of the carrier in question. Synonymous variants, meanwhile, alter the nucleotide without changing the encoded amino acid; they are usually considered to have a minimal impact on the function of the protein product, unless this change interferes with splicing regulation, such as the c.641A>G variant in *BRCA1* and the c.859G>A variant in *BRCA2*.

## Methods

### DNA Isolation and NGS Test

Genomic DNA was isolated from 500 µl of peripheral blood using the QIAsymphony SP system (QIAGEN, Germany) following the manufacturer’s instructions. The concentration and purity of the resulting DNA was determined by Qubit dsDNA HS assay (Life Technologies, USA) and NanoDrop 2000 UV-Vis Spectrophotometer (Thermo Scientific, USA), respectively. Library construction was performed as previously described using 200 ng of genomic DNA. The DNA was first fragmented into segments of approximately 300 bp by sonication (Diagenode, USA). Then appropriate sizing and quantification of the fragments was evaluated by the Agilent 2100 Bioanalyzer (Agilent Technologies, USA). The fragments were blunt-end-repaired and A-tailed to allow ligation of adapters, followed by PCR amplification. Enrichment of fragments covering 45 breast/ovarian cancer related genes was achieved using a probe set (Novogene, China) that captures a 0.26-Mb genomic region. The enriched library was sequenced on a NextSeq550 sequencer (Illumina, USA) generating paired end reads of 150 bp to a targeted coverage of over 500 unique reads.

### Variants Calling and Pathogenicity Assessment

Raw sequencing reads were cleaned and aligned to a human reference genome (GRCh37) using BWA (v0.7.17). SNVs and small insertions and deletions (indels) were called using the Genome Analysis Toolkit, Haplotype (v 4.1.7.0), freebayes (v 1.1.0.46), and SAMtools (v1.9). Then the called variants were annotated using snpEff (v4.3.1t). The pathogenicity of the detected variants was independently evaluated by two clinical geneticists according to the ACMG and ENIGMA (v 2.5.1) criteria. Annotations of the variants followed the Human Genome Variant Society recommendations.

### RNA Isolation, Reverse Transcription-Polymerase Chain Reaction, and Fragment Analysis

Total RNA was extracted and purified from 500 µl of peripheral blood using the EZ-press RNA Purification Kit PLUS (EZBioscience, China) according to the manufacturer’s instructions. Quantification and qualification of the isolated RNA were carried out using the NanoDrop 2000 UV-Vis Spectrophotometer (Thermo Scientific, USA). Reverse transcription was conducted to generate cDNA using 200ng of total RNA using HiScript II Q RT SuperMix (Vazyme, China), followed by PCR amplification using a pair of primers flanking the exon3 of *BRCA1* (F′-AGAGTGTCCCATCTGTCTGGA, R′- AAAGGACACTGTGAAGGCCC). Fragment analysis of the amplicon was performed using the Agilent 2100 Bioanalyzer (Agilent Technologies, USA).

### Minigene Splicing Assay

Wild type and mutant DNA fragments containing the entire exon and its flanking sequence of ±150 bp were synthesized by Sangon China. Then KpnI and BamHI sites were introduced at both ends of the sequence to insert into the pCAS2 vector as previously reported (8). The wild type and mutant vectors were transfected into HEK293T cells. After 24 hours of culturing, RNA was isolated from the transfected cells followed by RT-PCR using the following primers: F-TGACCCTGACCCCCCCT, R-TAAGGGCGATGCGAA. Then the amplicon was separated by gel electrophoresis for Sanger sequencing.

## Results And Discussion

### Detection of *BRCA1*:c.132C>T in Two Individuals From Unrelated Families

**Figure 1:**
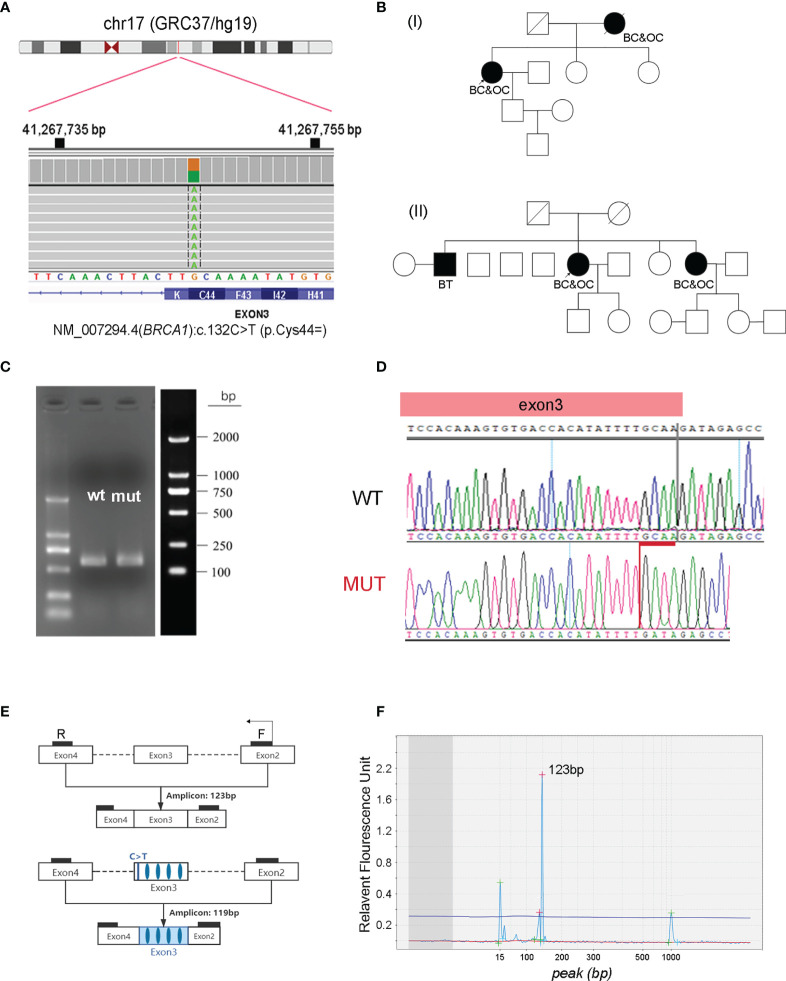
Characterization of NM_007294.4 (*BRCA1*):c.132C>T (p.Cys44=) as a pathogenic variant. **(A)** Identification of the heterozygous *BRCA1*:c.132C>T variant in one of the patients. The screen shot of this variant in the Integrative Genomics Viewer is presented. **(B)** Pedigrees of two unrelated patients carrying the synonymous *BRCA1*:c.132C>T variant. **(C)** PCR amplicons generated from wild-type (wt) and *BRCA1*:c.132C>T mutant (mut) constructs by a minigene assay, followed by agarose gel electrophoresis and band analysis. **(D)** Sanger sequencing determination of the deletion of GCAA (red) at the end of exon three in *BRCA1*:c.132C>T mutant cells. **(E)** Designed primers flanking exon three for RT-PCR amplification. **(F)** Fragment analysis of the RT-PCR product using RNA isolated from the blood cells of patients with *BRCA1*:c.132C>T variant. The amplicon size is evaluated by using the high-resolution automated electrophoresis on an Agilent 2100 Bioanalyzer. BC, breast cancer; OC, ovarian cancer; BT, brain tumor.

The first case was a 56-year-old female patient who was diagnosed with serous ovarian cancer (stage II) in the Affiliated Cancer Hospital of Zhengzhou University in 2018. The patient underwent a total abdominal hysterectomy with a lymph node dissection, followed by six cycles of carboplatin plus paclitaxel with a standard-dose regimen. Genetic testing identified a synonymous germline variant of NM_007294.4 (*BRCA1*):c.132C>T (p.Cys44=) (**Figure 1A**) in this patient, which was first interpreted as a VUS but could not exclude the possibility of being likely pathogenic. Genetic counseling revealed that the patient had undergone a mastectomy six years ago because of breast cancer, and that her mother experienced a similar history: breast cancer first followed by subsequent ovarian cancer (**Figure 1B**). Fortunately, the disease has been maintained in a stable state for several years, including at the latest review in May 2021.

The second case was a 64-year-old female patient, who was first diagnosed with breast cancer at 60 years of age, and then presented with ovarian cancer (stage IIIc) 4 years later in 2018. The patient followed a similar treatment as the case presented above: surgical resection followed by six cycles of carboplatin plus paclitaxel and three cycles of pegylated liposomal doxorubicin (PLD) plus nedaplatin. The disease was maintained in a stable state until January 2021, and the patient survived after local treatment. This patient was found to harbor the same germline variant of NM_007294.4 (*BRCA1*):c.132C>T (p.Cys44=). It was confirmed that one of her sisters, who is a carrier of the same variant, also developed breast cancer and ovarian cancer in her sixties (**Figure 1B**).

### Synonymous *BRCA1*:c.132C>T (p.Cys44=) Variant Results a Truncated Protein Product

This synonymous *BRCA1*:c.132C>T (p.Cys44=) variant is located in the 3^rd^ coding exon. It features a C to T substitution that occurs at nucleotide position 132 of the *BRCA1* gene. It has been recorded in the ClinVar database as having “conflicting interpretations of pathogenicity” (variation ID: 230061), with four submitted interpretations (two are of uncertain significance; the others are pathogenic). Observing these two independent carriers of *BRCA1:*c.132C>T prompted us to clarify the pathogenicity of this variant. This substitution occurs 3 bp from the end of exon two and does not change the amino acid at codon 44 (**Figure 1A**), in silico prediction (SSF, MaxEnt, NNSPLICE and GeneSplicer) suggests that this change may result in a truncated protein product of p.C44Yfs*5 (**Supplementary Figures 1**, **2**). Therefore, we first used the minigene assay to clarify if splicing was influenced by this synonymous variant, which revealed that this change caused the loss of the last four nucleotides (GCAA) in exon three (**Figures 1C, D**), consistent with a previous report by Steffensen et al. (17). We then isolated ribonucleic acid (RNA) from blood cells, followed by reverse transcription-polymerase chain reaction (RT-PCR) amplification using a pair of primers flanking exon three (**Figure 1E**). Fragment analysis using the Agilent 2100 bioanalyzer confirmed two peaks next to each other: a strong peak that was 123 bp long and a weak peak that was a few bp shorter (**Figure 1F**). This weak peak was probably caused by nonsense-mediated mRNA decay. Collectively, functional studies, in combination with the family history of these two independent carriers, convinced us to classify this synonymous *BRCA1*:c.132C>T (p.Cys44=) as a pathogenic variant.

### VUS and Synonymous Variants Reported in Databases

Female individuals carrying germline variants in *BRCA1/2* (*gBRCA1/2^MUT^*) have been well documented to have increased lifetime risk of developing multiple cancers. For ovarian cancer, their cumulative risk up to an age of 80 years increases to 44% for *gBRCA1^MUT^* and to 17% for *gBRCA2^MUT^* carriers, respectively (18). The frequencies and spectrums of *gBRCA1/2^MUT^* vary dramatically between different geographical regions and ethnic groups, ranging from 41% in Ashkenazi Jews to 13.8% in Americans (19). Several studies have shown that approximately 23–28% of Chinese Han OC patients present pathogenic or likely pathogenic *gBRCA1/2^MUT^*. In addition, these variants are distributed throughout the whole coding sequence, as well as in the flanking splicing regions, without any “hot spot” (20–22).

**Figure 2:**
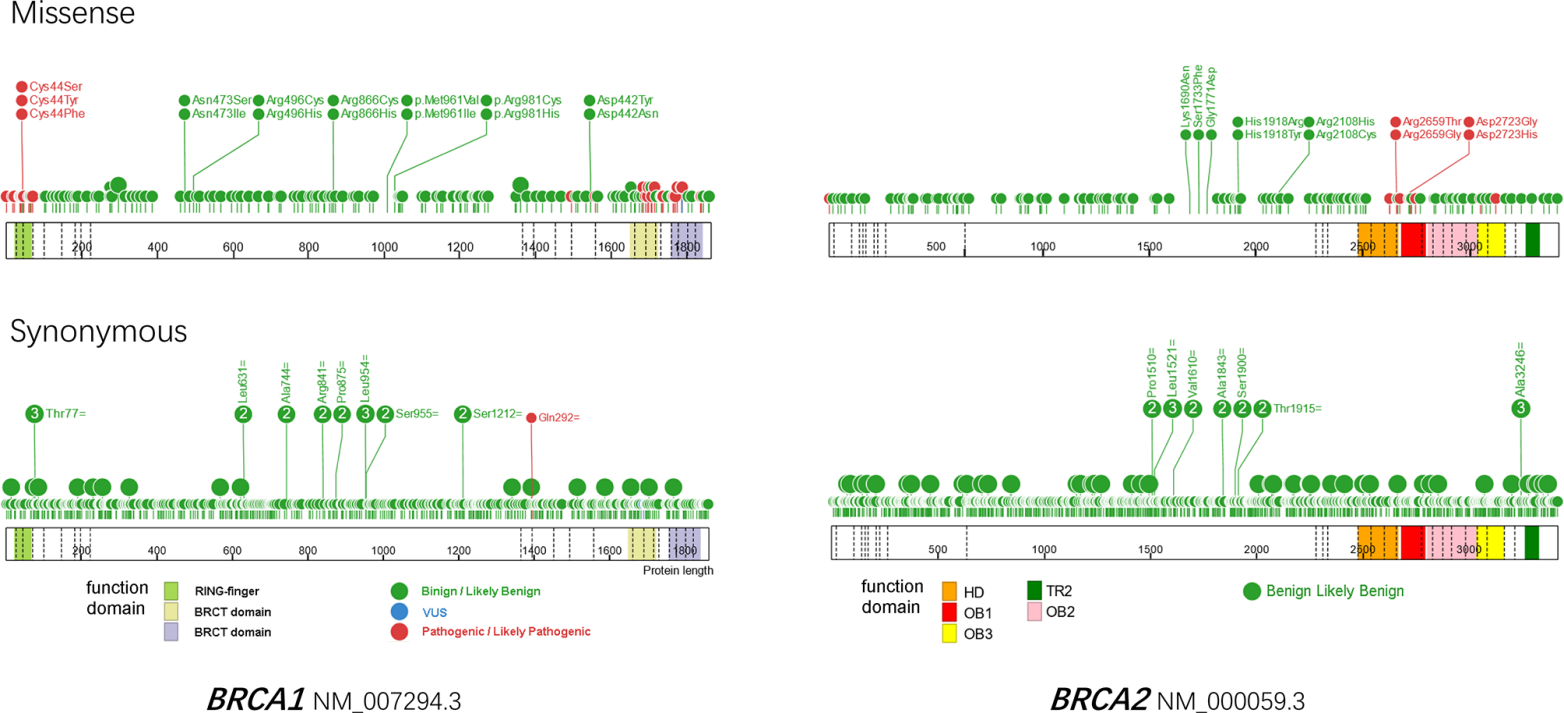
Protein paint of missense (upper) and synonymous variants reviewed by ENIGMA expert panel in *BRCA1* (left) and *BRCA2* (right). A total of 173 missense and 462 synonymous variants are plotted for *BRCA1*, as well as 143 missense and 782 synonymous variants for *BRCA2*. The reference transcripts of NM_007294.3 and NM_000059.3 are used for BRCA1 and BRCA2 respectively. Benign and likely benign variants are in green, VUS are in blue, pathogenic and likely pathogenic variants are in red. Dashed lines indicate exons. HD, helical domain; OB, oligosaccharide-binding folds; TR2, C-terminal RAD51 interaction domain.

Medical professionals have suggested that the *gBRCA1/2 ^MUT^* status in OC patients should be clarified as early as possible following their first diagnosis (23) because of its profound impact on treatment decisions and disease management. Both the Food and Drug Administration and European Medicines Agency have approved PARP inhibitors for the treatment of germline *BRCA^MUT^*-associated ovarian cancer. However, it remains challenging to correctly classify a new *BRCA1/2* variant in routine clinical practice. Approximately 10–20% of patients are reported to harbor *BRCA1/2* variants of unknown significance, which introduces uncertainty to their clinical management (24). We analyzed all of the submitted variants in the BRCA Exchange database (accessed in August 2021, https://brcaexchange.org) and found that in total, 38.63% (8355/21627) of the variants were classified as being of unknown significance, of which 92.75% (7749/8355) were missense and 7.25% (606/8355) were synonymous. Plotting the locations of expert-reviewed missense variants revealed a preference for pathogenic or likely pathogenic aberrations in the functional domains of *BRCA1* and *BRCA2*; almost all of the synonymous variants were benign or likely benign (**Figure 2**).

Synonymous variants are changes in a DNA sequence that do not change the encoded amino acid. Therefore, they are considered to be “silent”. However, if a synonymous variant disrupts splicing, either by creating a cryptic splice site or by interrupting the splicing regulatory elements, it could potentially abrogate protein function (25). Synonymous variants, as the “sound of silence”, is one of the most mysterious fields in the interpretation of *BRCA1/2* variants. Several *in silico* tools have been developed to predict the splicing changes caused by genetic variants (**Supplementary Table 1**), which is the most readily and commonly used approach (26–33).

Jian et al. compared the efficiency of these tools in predicting splicing defects by evaluating SNVs, without affecting the GT-AG dinucleotides at the 5′ and 3′ splice sites. They found that MaxEntScan and PWM outperformed other tools (9). This result was also confirmed by Houdayer et al. in *BRCA1* and *BRCA2* (34). The usDSM method, which uses 14-dimensional biology features and a random forest classifier, has been applied to achieve a superior performance in detecting deleterious synonymous variants. The deep learning model did not make a substantial contribution to the prediction, however, probably because of the limited training dataset used in the study (35). In addition to *in silico* prediction, the splicing reporter minigene assay is an efficient approach for evaluating the impact of an unclassified variant on mRNA splicing (36). It has been widely used in many laboratories worldwide. In this method, the variant of interest, along with its flanking intronic sequence (~150 bp) is PCR-amplified and cloned into the plasmid, followed by transient transfection into cultured cells. Then, RNA is isolated for reverse transcription, and the chimeric transcripts generated from both wild-type and mutant constructs are compared by PCR and sequencing to determine splicing changes. Several studies have shown that the impacts of variants on splicing patterns and protein functions are not always equivalent; they can vary depending on the proportions of truncated and functional isoforms (37–40).

**Figure 3:**
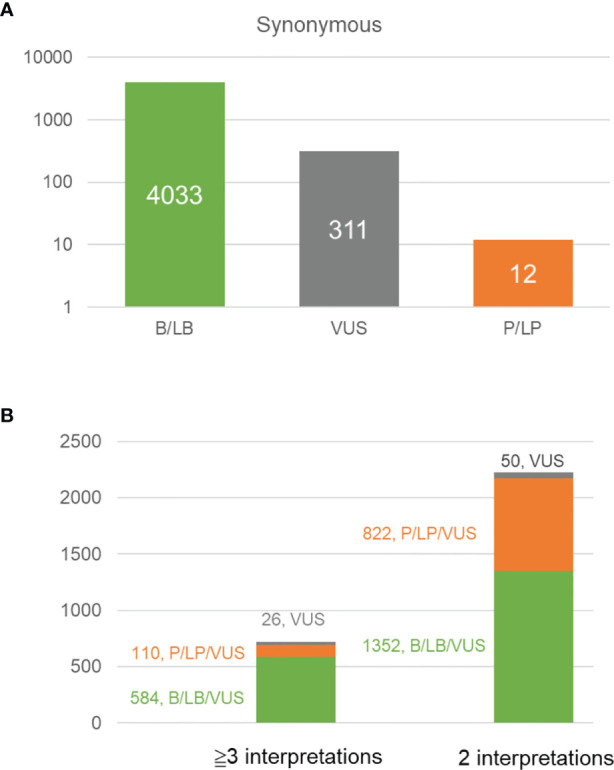
Analysis of the variants in different classes recorded in BRCA exchange database (accessed in Aug. 2021, https://brcaexchange.org). **(A)** Bar plot showing the number of benign/likely benign variants (4033, in green), VUS (311, in gray), and pathogenic/likely pathogenic variants (12, in orange) in BRCA exchange database. **(B)** Bar plot shows the number of variants with different interpretations in the BRCA exchange database. Green, the variant with different interpretations of benign, likely benign or VUS; orange, the variant with different interpretations of pathogenic, likely benign and VUS; gray, the variants of VUS.

We analyzed all synonymous variants in the BRCA exchange database. Unsurprisingly, 92.6% (4033/4356) of the variants were consistently classified as being benign or likely benign, 7.1% (311/4356) were VUS, and only 0.3% (12/4356) were pathogenic or likely pathogenic (**Figure 3A**). Furthermore, discordant interpretations were more frequently observed in benign/likely benign/VUS variants than in pathogenic/likely pathogenic/VUS ones (**Figure 3B**). In total, ten synonymous variants were classified as being pathogenic or likely pathogenic by at least one submitter (**Supplementary Table 2**). By reviewing these variants, we interpreted c.4992C>T (p.Leu1664=) in *BRCA1* (NM_007294.4) as benign, *BRCA1*:c.5022C>T (p.Ile1674=) and *BRCA2*:c.7992T>A (p.Ile2664=) as likely benign, and c.5277G>A (p.Lys1759=) in *BRCA1* and c.9117G>T (p.Pro3039=) in *BRCA2* as VUS (due to insufficient evidence). The other variants were classified as being either pathogenic or likely pathogenic. One common feature of pathogenic or likely pathogenic variants with synonymous changes is that they always occur at the end of an exon. This suggests that particular caution is warranted regarding the variants in this region. If only *in silico* predictions of splicing changes are to be used as evidence, without functional and RNA confirmation, then these variants could only be classified as VUS. As mentioned above, the proportions of dysfunctional isoforms caused by aberrant splicing also determines the eventual biological consequences. For example, the minigene assay showed that *BRCA1*: c.557C>A results in an isoform with excluded exons. However, the carrier’s RNA analysis revealed only a minor proportion of this aberrant isoform; therefore, this variant was still considered to be a VUS. It is worth noting that *BRCA1*: c.557C>A was not predicted to cause aberrant splicing by several *in silico* tools, probably because of its location in the internal exon. Some synonymous variants that occur in exons, such as NM_000059.3 (*BRCA2*):c.9057A>G (p.Lys3019=), can induce aberrant splicing, but most bioinformatic tools cannot efficiently predict these culprits (41).

In addition to the canonical approaches to interpreting *BRCA1/2* variants, such as functional studies and multifactorial likelihood quantitative analysis, genome-wide association studies have identified many genetic variants associated with *BRCA1/2* expression levels and post-translational modifications. These variants are thereby associated with the risk of developing breast and ovarian cancer (42–44). Expression quantitative trait locus (eQTL) analysis is commonly used to interpret the transcription regulatory mechanisms of genetic variants, which can be either in *cis* (<1 Mb) or *trans* (>5 Mb or on another chromosome) (45). The single nucleotide polymorphisms (SNPs) rs17742929 and rs12952924 have been identified as *cis*-eQTL and *trans*-eQTL, respectively, for *BRCA1* expression in breast cancer. Furthermore, rs7988807 and rs277271 have been identified as *cis*-eQTL and *trans*-eQTL, respectively, for *BRCA2* expression (46). It has previously been shown that rs57025206 alone can serve as an independent survival marker for estrogen receptor negative *BRCA1^MUT^* breast cancer patients, so it can predict unfavorable outcomes (47). The SNPs rs56187033 and rs56012641 are in the post-translational modification sites of *BRCA1* and are associated with decreased phosphorylation and N-glycosylation, respectively. However, they were predicted by multiple *in silico* tools to be neutral, and they have been classified by an expert panel as benign variants (variation IDs 37661 and 37423, respectively). Other non-coding SNPs, such as rs799923, rs799916, and rs3092994, have also been predicted to affect transcription factor binding and have been shown to be circular RNA binding sites, leading to deleterious biological consequences (42). Future studies should pay more attention to these SNPs, as most of them are considered to be benign variants based on current American College of Medical Genetics and Genomics or ENIGMA guidelines but are likely to also cause deleterious clinical outcomes.

## Conclusion

*BRCA1* and *BRCA2* were identified and isolated in the 1990s by researchers from the United States of America and the United Kingdom, respectively. They were identified as breast cancer susceptibility genes, and their essential roles in maintaining genome stability and integrity by regulating DNA damage response and repair were revealed (48). Although many studies have focused on *BRCA1/2*, as Aristotle famously wrote: “the more you know, the more you know you don’t know”. Future studies should aim to clarify the clinical significance of remaining VUS, investigate the regulatory mechanism of *BRCA1/2* expression, and build connections between genetic variants with large-scale clinical data. These efforts could help to better understand these genes and related cancers.

## Data Availability Statement

The datasets presented in this study can be found in Genome Sequence Archive (GSA) database with the accession numbers of HRI102015 and HRI102067, further inquiries can be directed to the corresponding author.

## Ethics Statement

The studies involving human participants were reviewed and approved by the ethics committee of Henan Cancer Hospital. The patients/participants provided their written informed consent to participate in this study.

## Author Contributions

JL, PW, and HX conceived the idea and drafted the manuscript. CZ performed the bioinformatics analysis. SH and PW collected the patients’ medical records and performed the RT-PCR experiment. ZL carried out the minigene assay. XW, BW, and JM joined manuscript editing. HL and YG supervised and supported the study. All authors contributed to the article and approved the submitted version.

## Funding

JL was supported by the Henan provincial young researcher program. This work was financially supported by the funding from National Natural Science Foundation of China (grant number: 81802779), Henan Provincial Health Commission (grant number:SBGJ202002020), Henan science and technology project (grant numbers: 212102310675 and 212102310738), and major public welfare projects in Henan Province (grant number: 201300310400).

## Conflict of Interest

Author ZL is currently employed by Amoy Diagnostics Co., Ltd., Xiamen, China.

The remaining authors declare that the research was conducted in the absence of any commercial or financial relationships that could be construed as a potential conflict of interest.

## Publisher’s Note

All claims expressed in this article are solely those of the authors and do not necessarily represent those of their affiliated organizations, or those of the publisher, the editors and the reviewers. Any product that may be evaluated in this article, or claim that may be made by its manufacturer, is not guaranteed or endorsed by the publisher.
