# Supplementary material for: Characterization of Synonymous BRCA1:c.132C>T as a Pathogenic Variant
Source: Front Oncol. 2022 Jan 11;11:812656. doi: 10.3389/fonc.2021.812656 (PMC8789006; doi:10.3389/fonc.2021.812656)
Supplement: Supplementary file 2 [file Image_2.pdf]

Reference protein

```
   1 MDLSALRVEE VQNVINAMQK ILECPICLEL IKEPVSTKCD HIFCKFCMLK LLNQKKGPSQ
  61 CPLCKNDITK RSLQESTRFS QLVEELLKII CAFQLDTGLE YANSYNFAKK ENNSPEHLKD
 121 EVSIIQSMGY RNRAKRLLQS EPENPSLQET SLSVQLSNLG TVRTLRTKQR IQPQKTSVYI
 181 ELGSDSSEDT VNKATYCSVG DQELLQITPQ GTRDEISLDS AKKAACEFSE TDVTNTEHHQ
 241 PSNNDLNTTE KRAAERHPEK YQGSSVSNLH VEPCGTNTHA SSLQHENSSL LLTKDRMNVE
 301 KAEFCNKSKQ PGLARSQHNR WAGSKETCND RRTPSTEKKV DLNADPLCER KEWNKQKLPC
 361 SENPRDTEDV PWITLNSSIQ KVNEWFSRSD ELLGSDDSHD GESESNAKVA DVLDVLNEVD
 421 EYSGSSEKID LLASDPHEAL ICKSERVHSK SVESNIEDKI FGKTYRKKAS LPNLSHVTEN
 481 LIIGAFVTEP QIIQERPLTN KLKRKRRPTS GLHPEDFIKK ADLAVQKTPE MINQGTNQTE
 541 QNGQVMNITN SGHENKTKGD SIQNEKNPNP IESLEKESAF KTKAEPISSS ISNMELELNI
 601 HNSKAPKKNR LRRKSSTRHI HALELVVSRN LSPPNCTELQ IDSCSSSEEI KKKKYNQMPV
 661 RHSRNLQLME GKEPATGAKK SNKPNEQTSK RHDSDTFPEL KLTNAPGSFT KCSNTSELKE
 721 FVNPSLPREE KEEKLETVKV SNNAEDPKDL MLSGERVLQT ERSVESSSIS LVPGTDYGTQ
 781 ESISLLEVST LGKAKTEPNK CVSQCAAFEN PKGLIHGCSK DNRNDTEGFK YPLGHEVNHS
 841 RETSIEMEES ELDAQYLQNT FKVSKRQSFA PFSNPGNAEE ECATFSAHSG SLKKQSPKVT
 901 FECEQKEENQ GKNESNIKPV QTVNITAGFP VVGQKDKPVD NAKCSIKGGS RFCLSSQFRG
 961 NETGLITPNK HGLLQNPYRI PPLFPIKSFV KTKCKKNLLE ENFEEHSMSP EREMGNENIP
1021 STVSTISRNN IRENVFKEAS SSNINEVGSS TNEVGSSINE IGSSDENIQA ELGRNRGPKL
1081 NAMLRLGVLQ PEVYKQSLPG SNCKHPEIKK QEYEEVVQTV NTDFSPYLIS DNLEQPMGSS
1141 HASQVCSETP DDLLDDGEIK EDTSFAENDI KESSAVFSKS VQKGELSRSP SPFTHTHLAQ
1201 GYRRGAKKLE SSEENLSSED EELPCFQHLL FGKVNNIPSQ STRHSTVATE CLSKNTEENL
1261 LSLKNSLNDC SNQVILAKAS QEHHLSEETK CSASLFSSQC SELEDLTANT NTQDPFLIGS
1321 SKQMRHQSES QGVGLSDKEL VSDDEERGTG LEENNQEEQS MDSNLGEAAS GCESETSVSE
1381 DCSGLSSQSD ILTTQQRDTM QHNLIKLQQE MAELEAVLEQ HGSQPSNSYP SIISDSSALE
1441 DLRNPEQSTS EKAVLTSQKS SEYPISQNPE GLSADKFEVS ADSSTSKNKE PGVERSSPSK
1501 CPSLDDRWYM HSCSGSLQNR NYPSQEELIK VVDVEEQQLE ESGPHDLTET SYLPRQDLEG
1561 TPYLESGISL FSDDPESDPS EDRAPESARV GNIPSSTSAL KVPQLKVAES AQSPAAAHTT
1621 DTAGYNAMEE SVSREKPELT ASTERVNKRM SMVVSGLTPE EFMLVYKFAR KHHITLTNLI
1681 TEETTHVVMK TDAEFVCERT LKYFLGIAGG KWVVSYFWVT QSIKERKMLN EHDFEVRGDV
1741 VNGRNHQGPK RARESQDRKI FRGLEICCYG PFTNMPTDQL EWMVQLCGAS VVKELSSFTL
1801 GTGVHPIVVV QPDAWTEDNG FHAIGQMCEA PVVTREWVLD SVALYQCQEL DTYLIPQIPH
1861 SHY*
```

Protein predicted from variant coding sequence

```
1 MDLSALRVEE VQNVINAMQK ILECPICLEL IKEPVSTKCD HIFYFAC*
```

Supplementary Figure 2. Truncated protein predicted to generated from *BRCA1*:c.132C>T variant. Reference protein sequence of BRCA1 is shown in the upper section that the amino acids influenced by this change are marked in red. The predicted protein sequence from the variant coding sequence is shown in the lower panel that a premature stop codon is identified four amino acids afterward.
