# Supplementary material for: Characterization of Synonymous BRCA1:c.132C>T as a Pathogenic Variant
Source: Front Oncol. 2022 Jan 11;11:812656. doi: 10.3389/fonc.2021.812656 (PMC8789006; doi:10.3389/fonc.2021.812656)
Supplement: Supplementary file 3 [file Table_1.docx]

Supplementary Table 1. *In silico* tools used to predict splicing changes.

| *In silico* tool | Prediction | Access |
| --- | --- | --- |
| MaxEntScan | Splicing *cis* elements that have been reported | <http://www.umd.be/HSF3/> |
| HumanSplicingFinder, SliceSiteFinder-like | Splice site detection using position weight matrices (PWMs) | <https://www.genomnis.com/> |
| NNSplice | Neural network-based prediction of splice sites | <https://fruitfly.org/seq_tools/splice.html> |
| GeneSplicer | Splice site prediction using a combination of Markov and MDD models | <http://www.ccb.jhu.edu/software/genesplicer/> |
| Spliceman | Position distribution of the variants in splicing elements | <http://fairbrother.biomed.brown.edu/spliceman/> |
| Skippy | Exonic variants that modulate splicing | <https://research.nhgri.nih.gov/skippy/> |
| SPANR | Exonic and intronic single nucleotide variants (SNVs) that are up to 300 nt from splice junctions | <http://tools.genes.toronto.edu/> |

Supplementary Table 2. Consensus of pathogenic or likely pathogenic synonymous variants in the BRCA exchange database.

| **INFO** | **Classification**  **in BRCA Exchange** | **Expert**  **panel** | **Author’s**  **review** | **Location** | ***In silico* prediction** | **RNA**  **Analysis** | **Minigene**  **assay** | **Functional evidence** | **Clinical observation** |
| --- | --- | --- | --- | --- | --- | --- | --- | --- | --- |
| *BRCA1*:c.132C>T (p.Cys44=) | VUS/P | ~ | P | Third nucleotide at the end of exon 3 | Yes | Yes | Yes | Yes | Yes |
| *BRCA1*:c.4185G>A（p.Gln1395=) | P | P | P | Last nucleotide of exon 11 | Yes | Yes | Yes | Yes | Yes |
| *BRCA1*:c.4992C>T (p.Leu1664=) | P/VUS/LB/B | B | B | In the middle of exon 16 | - | - | - | - | - |
| *BRCA1*:c.5022C>T (p.Ile1674=) | LB/VUS/P | LB | LB | In the middle of exon 16 | - | - | - | - | - |
| *BRCA1*:c.5277G>A (p.Lys1759=) | VUS/P | ~ | VUS | Last nucleotide of exon 19 | Conflicting | No | No | No | Yes |
| *BRCA2*:c.516G>A (p.Lys172=) | VUS/P/LP | ~ | LP | Last nucleotide of exon 6 | Yes | Yes | Yes | Yes | Yes |
| *BRCA2*:c.7992T>A (p.Ile2664=) | B/LB/VUS/LP | LB | LB | 16^th^ nucleotide at the end of exon 18 | - | - | - | - | - |
| *BRCA2*:c.8754G>A (p.Glu2918=) | P/LP | ~ | P | Last nucleotide of exon 21 | Yes | Yes | Yes | Yes | Yes |
| *BRCA2*:c.9057A>G (p.Lys3019=) | VUS/P/LP | ~ | LP | In exon 23 (60 nt) | Yes | Yes | No | Yes | Yes |
| *BRCA2*:c.9117G>T (p.Pro3039=) | VUS/P | ~ | VUS | Last nucleotide of exon 23 | Yes | No | No | No | Yes |
| *BRCA2*:c.9117G>A (p.Pro3039=) | P | ~ | P | Last nucleotide of exon 23 | Yes | Yes | Yes | Yes | Yes |

Note: ~, not included, B or LB variants with solidate evidence that don’t have conflicting interpretations.
