# Supplementary material for: Characterization of Synonymous BRCA1:c.132C>T as a Pathogenic Variant
Source: Front Oncol. 2022 Jan 11;11:812656. doi: 10.3389/fonc.2021.812656 (PMC8789006; doi:10.3389/fonc.2021.812656)
Supplement: Supplementary file 1 [file Image_1.pdf]

A

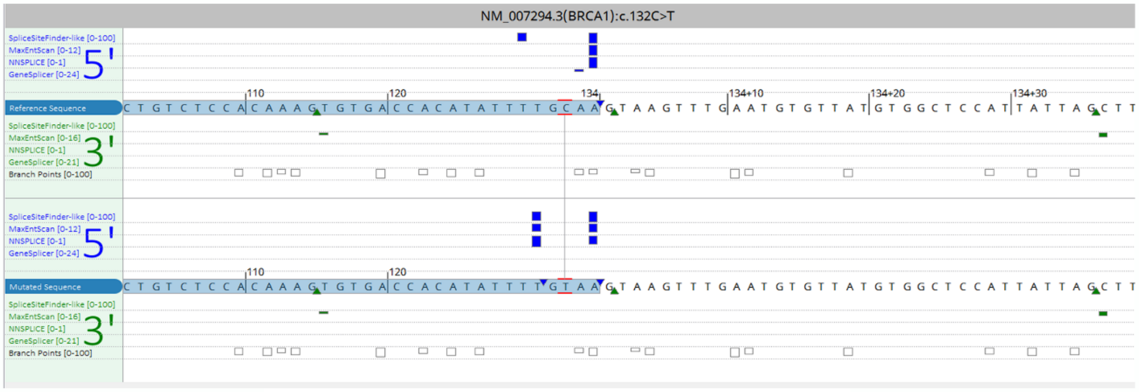

B

Gene: **BRCA1** - Transcript: **NM_007294.3** - Variant: **c.132C>T**
Analysis range: **c.134+38 (Intron 3) - c.92 (Exon 3) [81 bps]**

Donor Sites

	SSF [0-100]	MaxEnt [0-12]	NNSPLICE [0-1]	GeneSplicer [0-24]
Threshold	≥ 70	≥ 0	≥ 0.4	≥ 0
Exon 3 - c.129	71.22⇒ —			
Exon 3 - c.130	— ⇒ 74.75	— ⇒ 7.40	— ⇒ 0.90	
Exon 3 - c.133				2.61⇒ —
Exon 3 - c.134 ℕ	87.86=> 83.74 (-4.7%)	10.08=> 8.07 (-20.0%)	0.89=> 0.70 (-21.9%)	

ℕ: Natural Splice Site

Acceptor Sites

	SSF [0-100]	MaxEnt [0-16]	NNSPLICE [0-1]	GeneSplicer [0-21]
Threshold	≥ 70	≥ 0	≥ 0.4	≥ 0
Exon 3 - c.115		= 2.65		
Intron 3 - c.134+36		= 4.39		

Supplementary Figure 1. In silico prediction of *BRCA1*:c.124C>T in splicing changes. Synonymous *BRCA1*:c.124C>T variant is predicted to alter splicing by deleting 4bp at the end of exon3 of BRCA1 **(A)**, and the scores given by different in silico tools are shown in **(B)**.
